# Modeling and Evaluation of Ash-Forming Element Fate and Occurrence in Woody Biomass Combustion in an Entrained-Flow Burner

**DOI:** 10.1021/acsomega.1c06445

**Published:** 2022-05-04

**Authors:** Wahyu Meka, Janos Szuhanszki, Karen Finney, Bijal Gudka, Jenny Jones, Mohamed Pourkashanian, Paul S. Fennell

**Affiliations:** † Department of Chemical Engineering, Imperial College London, South Kensington Campus, London SW7 2AZ, England, United Kingdom; ‡ Energy 2050, Department of Mechanical Engineering, University of Sheffield, Sheffield S1 3JD, United Kingdom; § School of Chemical and Process Engineering, Faculty of Engineering, University of Leeds, Leeds LS2 9JT, United Kingdom

## Abstract

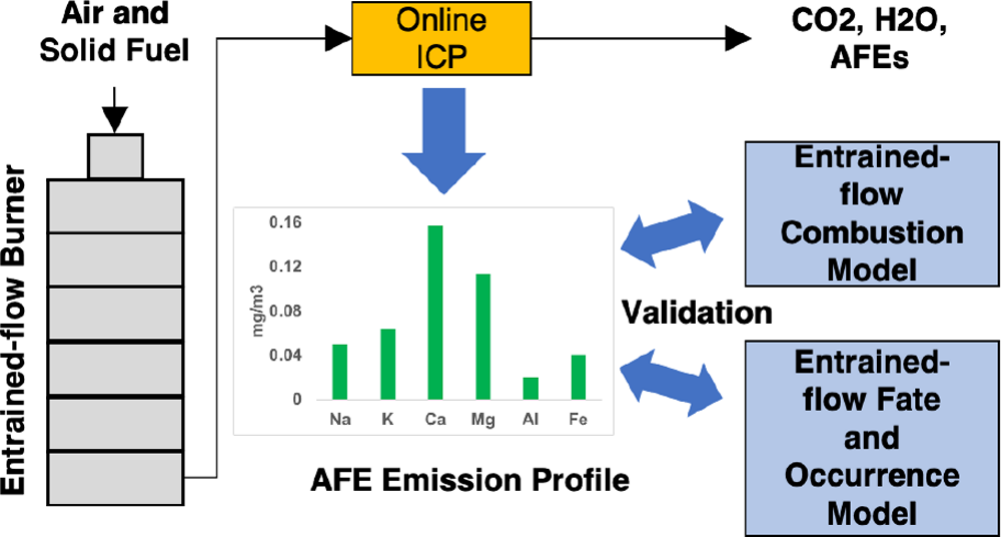

Biomass combustion equipment is often susceptible to ash deposition due to the relatively significant quantities of potassium, silicon, and other ash-forming elements in biomass. To evaluate the propensity for ash deposition resulting from biomass combustion, a biomass combustion model was integrated with a chemical equilibrium model to predict the fate and occurrence of ash-forming elements in a pilot-scale entrained-flow burner. The integrated model simulated the combustion of white wood (virgin wood) and recycled wood (treated wood) previously combusted in the burner. The key advantage of this model in comparison to a model with general equilibrium assumed is that it was able to consider the rate of release of trace and minor species with time, the local equilibrium in the particles, and separately, that in the continuum phase (which also included any solid or liquid materials nucleating). The simulation generated the fate and occurrence profiles of each ash-forming element along the burner. The qualitative comparisons between the modeled profiles and the previous experimental findings under similar operating conditions show reasonable agreement. The concentrations of ash-forming elements released from the burner were also compared with the experimental online inductively coupled plasma readings. However, the latter comparison shows overestimation using the modeled results and might suggest that further considerations of other parameters such as ash nucleation and coagulation are required. Nonetheless, based on the ongoing performance of the integrated model, future use of the model might be expanded to a broader range of problematic solid fuels such as herbaceous biomass or municipal solid waste.

## Introduction

The global energy demand is predicted to grow by more than twice its current value by 2040. Unless there is a change in future energy policies, this growth will contribute to a global energy-related CO_2_ emission increase of 10 Gt.^1^ This emission increase is corroborated by a study revealing that there was a 2.7% increase of global carbon emission between 2015 and 2018 due to intense coal and oil utilization for driving economic growth in China and India.^2^

Coal replacement by biomass in solid fuel combustion is one option to reduce global carbon emission. Biomass combustion features no net CO_2_ addition to the atmosphere since the released carbon originates from the CO_2_ absorbed by the plants that are the precursors for biomass. Biomass combustion also promotes reduced environmental pollution since biomass has lower sulfur, nitrogen, and heavy metal content than coal.^3^

Biomass combustion also offers advantages in reactivity and burnout behavior. Biomass is more reactive than coal, and biomass ignition starts at a relatively lower temperature.^4^ Low-temperature ignition reduces combustion delay time and establishes stable flames. A previous study also showed that biomass has high conversion due to significant volatile matter content.^5^

However, biomass combustion potentially causes more severe operational problems than coal combustion. Biomass ash contains significant quantities of ash-forming elements (AFEs) and chlorine. Alkali AFE reactions with chlorine produce liquid alkali chloride aerosols, which can induce fouling and corrosion on metal surfaces in commercial boiler convective pass zones. Silicon and calcium reactions with O_2_ produce SiO_2_ and calcium silicate-type compounds, which can deposit on commercial boiler water walls and reduce heat transfer efficiency. Alkali chloride deposition rates are enhanced when they further react with SiO_2_ by forming sticky liquid alkali silicate aerosols.

**Table 1:** Operating Conditions at the PACT Entrained-Flow Burner

parameter	value
inlet air flow rate (mol s^–1^)	2.79
inlet gas temperature (K)	480
inlet white wood flow rate (g s^–1^)	11.1
inlet recycled wood flow rate (g s^–1^)	11.7

**Table 2:** Characterization of Modeled Biomass

analysis	component	white wood	recycled wood
proximate analysis (% a.r. mass)	moisture	6.69	5.80
	volatile matter	78.10	73.90
	fixed carbon	14.51	17.10
	ash	0.70	3.20
ultimate analysis (% a.r. mass)	C	48.44	51.90
	H	6.34	6.00
	O	37.69	41.70
	N	0.15	0.40
	S	0.02	0.02
	Cl	0.01	0.01
AFEs (a.r. mass ppm in fuel)	Na	62.99	335.44
	K	547.59	650.62
	Ca	1260.27	6355.36
	Mg	216.28	745.53
	Si	415.28	6257.57
	Al	65.67	925.28
	Fe	59.39	1602.47
	P	N/A	N/A
	Ti	N/A	N/A

Enhanced ash sticking probabilities due to liquid phase formation cause several operational problems. Accumulation of ash deposits on heat exchanger pipe surfaces is known to reduce heat transfer rates and overall plant efficiencies.^6^ The growth of the ash deposits could stretch to neighboring heat exchanger pipes and block gas flows.^7^ Reduced gas flow rates create local pressure and temperature buildup, causing damage to the heat exchanger pipes.^8^ The buildup of local temperatures enhances corrosion and further damages the heat exchanger pipe surfaces. Sticky biomass ash also forms agglomerations in fluidized-bed boilers. Agglomerations create channels and reduce combustion efficiencies by inhibiting bed fluidization.^9^ Unplanned plant shutdowns are often mandatory to facilitate the removal of the accumulated ash deposits.^10^ Consequently, frequent shutdowns might adversely impact plant lifetimes due to thermal stresses.^11^

Chemical equilibrium is considered here as a potential tool to evaluate AFE phases in biomass combustion via predictions of the fate and occurrence of the AFEs. The use of chemical equilibrium in biomass combustion has been widely deployed to enable estimations with the absence of reaction kinetic parameters.^12−14^ Due to lack of reaction kinetic parameters and, to some extent, mass transfer effects, deviations between theoretical calculation results and experimental measurement results are anticipated.^15^

This paper aims to integrate a comprehensive steady-state combustion model with a chemical equilibrium model to work toward predicting the fate and occurrence of AFEs in biomass combustion. The validity of the predictions was evaluated via comparisons of the modeling results with previous findings and online measurements. The predictions are expected to lead to recommendations as to whether any examined fuel is safe to burn and whether additional treatment of the examined fuel is required to ensure an operationally safe combustion process.

## Experimental Section

**Figure 1:**
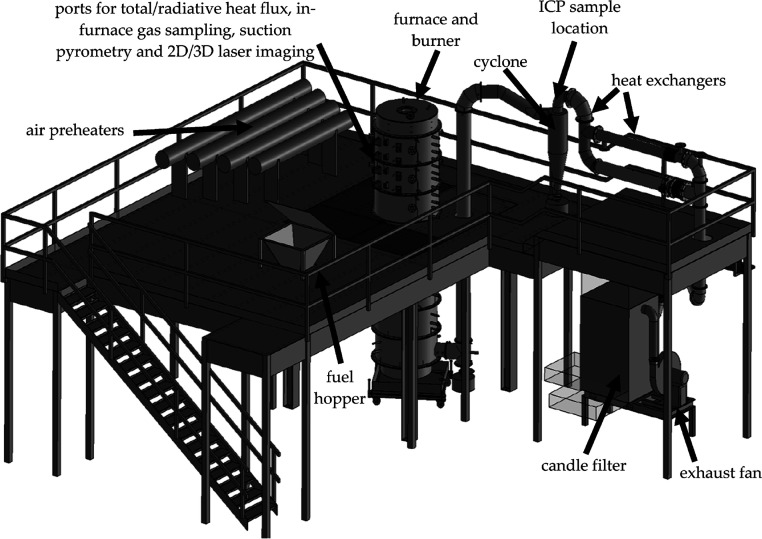
Three-dimensional diagram of the PACT facility 250 kW combustion unit. Reprinted in part with permission from ref (19). Copyright 2018 MDPI.

The 250 kW entrained-flow combustion unit (Figure 1) at the PACT facility in Sheffield was used to experimentally investigate the fate and occurrence of various elements during solid fuel combustion.^16−18^ Gas heaters consisting of primary, secondary, tertiary, and overfire heaters preheat the oxidizer before it enters the burner and combustion chamber. The oxidizer from the primary heater outlet is mixed with the solid pulverized fuel fed via the feeder, which is sent to the burner, with the flows from the tertiary and overfire heater outlets providing additional oxygen staged throughout the burner and reactor to encourage complete fuel burnout and minimize emissions. The scaled swirl burner was provided by GE. The cylindrical furnace is 4 m in height and has an internal diameter of 0.9 m. The burner column has eight axial sections, each measuring 0.5 m in length, with a 0.1 m gap made of thick refractories between adjacent sections. The solid fuel and oxidizer enter at the top of the burner column in a down-fired arrangement. Temperature profiles and gas concentration profiles are measured with probes installed along the burner centerline of the combustion chamber. The combustion gases leave the burner column at the bottom and then flow upward—the ash catch pot at the bottom of this section collects large ash particles, and the cyclone at the top removes smaller particles. The combustion gases then flow through the heat exchanger. After heat transfer in the heat exchanger, the combustion gases go through a candle filter to remove smaller particulates and, potentially, some aerosols before being released to the atmosphere via the stack. The operating conditions of the combustor are listed in Table 1. The same thermal input was used in both cases; due to the differences in the energy content between the fuels, the fuel flow rate of the recycled wood was slightly higher than for the white wood.

**Figure 2:**
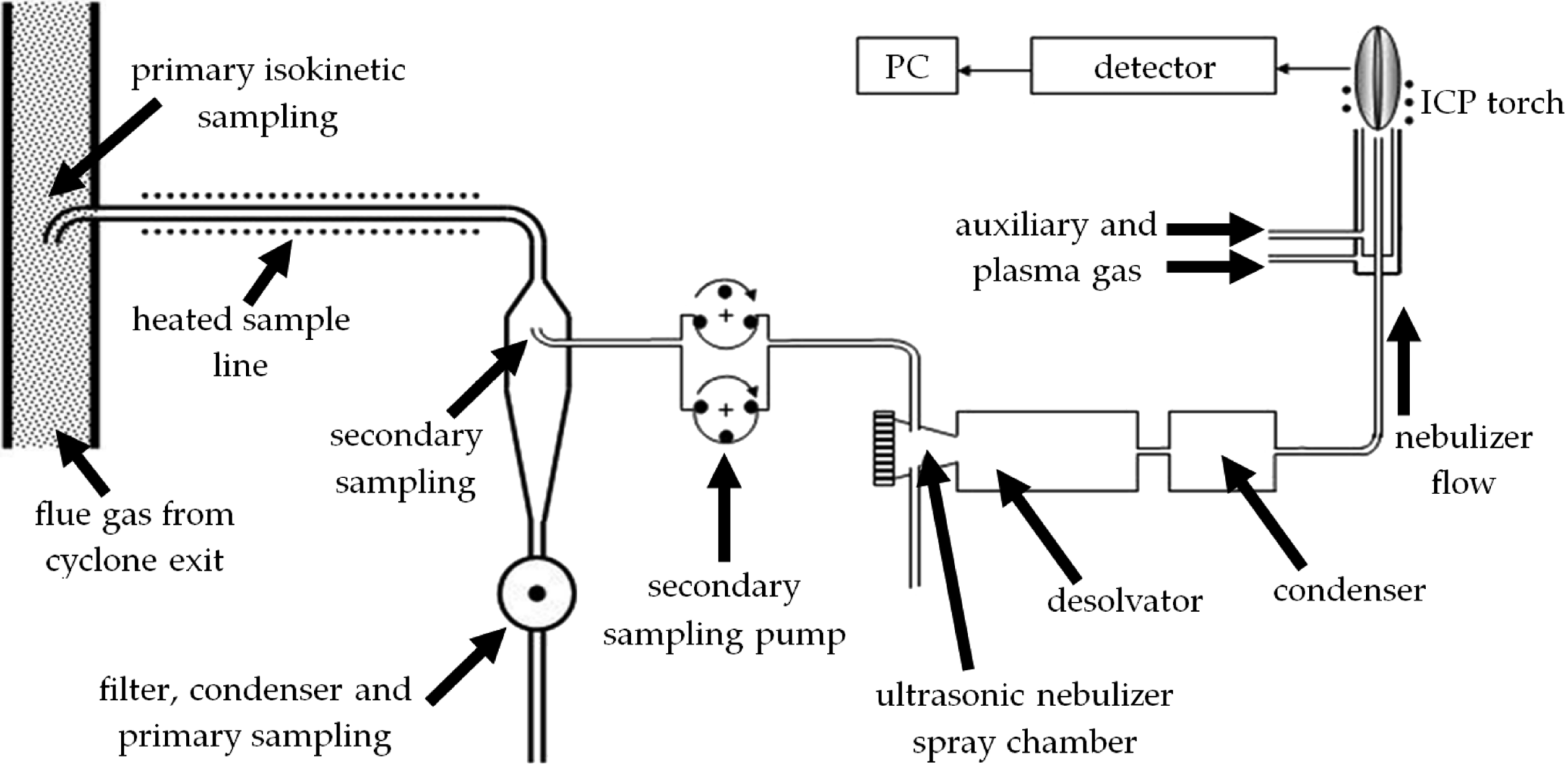
Flow diagram of PACT online ICP. Reprinted in part with permission from ref (19). Copyright 2018 MDPI.

A small fraction of the combustion gases leaving the cyclone flows are sampled for analysis with an online inductively coupled plasma (ICP) spectrometer; this determines the levels and species of combustion-generated entrained metal aerosol emissions. The combustion gases are pumped to the ICP at 200 mL/min with a peristaltic pump and heated in the heated sample line at 180 °C to avoid moisture loss. The combustion gases are then cooled in the desolvator and the condenser at 150 and 2 °C, respectively, to trap moisture (Figure 2). The dried combustion gases then enter the plasma flame at ∼6000 K. At this temperature, any previously formed aerosols are expected to completely vaporize and will be identified in the optical detector located radially to the torch. The instrument was calibrated according to the method outlined by Finney et al.^19^ This was conducted with solutions at a range of concentrations for the species investigated herein. The element standards and blank solution were used to form calibration curves for each element, where the average lower detection limit was 0.041 mg/m^3^ and the upper detection limit was 86.9 mg/m^3^. The correlation coefficients averaged 1.00.

The experimental measurement and the previous literature findings based on the combustion of white wood and recycled wood in the entrained-flow combustion unit were used in the evaluation and validation of the integrated model. The characteristics of both white wood and recycled wood are listed in Table 2. The contrasting ash quantities allow the evaluation of the influence of ash quantities on the fate and occurrence of AFEs.

## Theoretical Calculations

**Figure 3:**
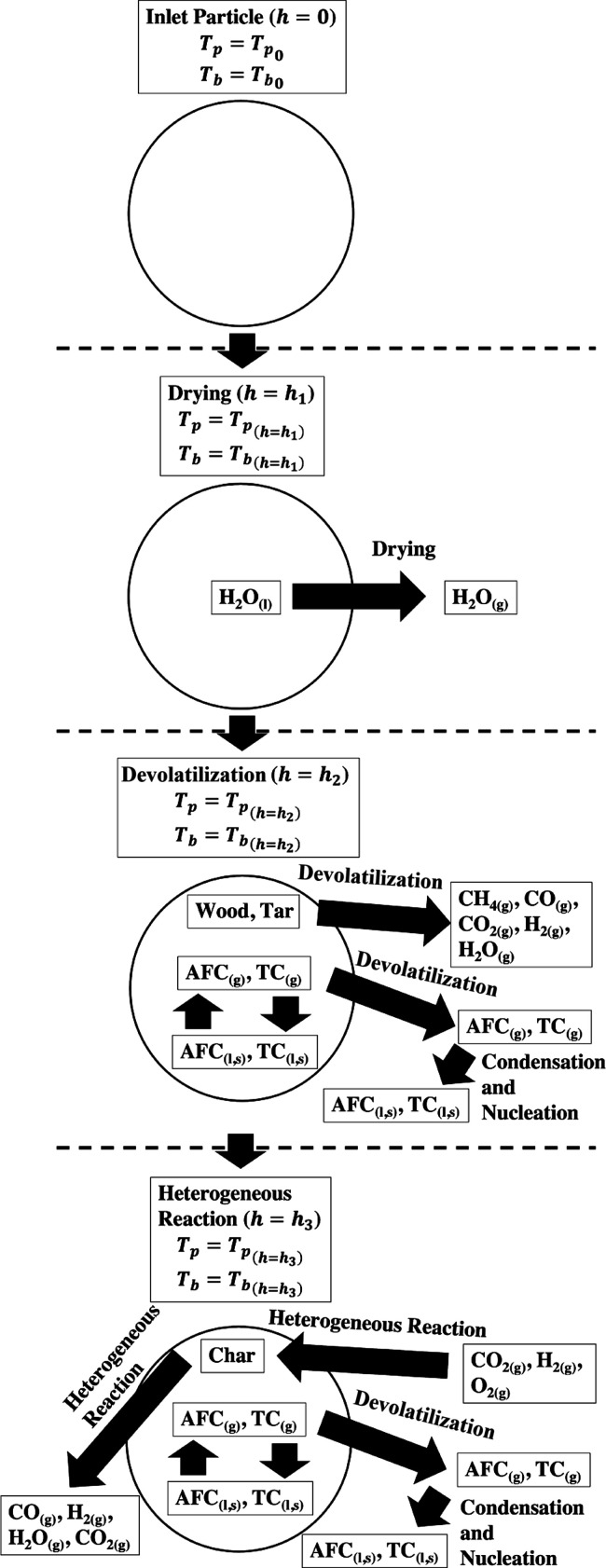
General fate of AFEs and TEs during the falling of a particle through a burner. Note that *h*_1_, *h*_2_, and *h*_3_ here are simply stages of combustion and not discretized heights.

Two separate models were developed, namely, the entrained-flow combustion model (ECM) and the entrained-flow fate and occurrence model (EFOM), based on compound fate and occurrence in Figure 3. The ECM modeled the one-dimensional entrained-flow combustion and generated the profiles of both bulk temperatures and bulk gas concentrations of major gases, e.g., CH_4_, CO, CO_2_, H_2_, H_2_O, and N_2_, in the entrained-flow burner. These profiles were used as parameters in the EFOM to calculate and generate the profiles of the fate and occurrence of each AFE in the entrained-flow burner using chemical equilibrium calculations.

The ECM and EFOM were developed within Matlab and ChemApp, respectively. ChemApp contains digital libraries of thermodynamic properties and allows calculations of the Gibbs free energies for an enormous number of chemical compounds. The digital libraries are without user interfaces and work as a foreign object of a chemical equilibrium function called by Matlab via a user-written bridging code. This calculation route provides the same results as that of the well-known equilibrium analysis software, Factsage, as a standalone program without excessive time consumption.

The ECM conducts simultaneous numerical calculations of bulk temperatures and bulk major gas concentration from the top to the bottom of the burner column. The burner column is discretized into thin slices. Each thin slice consists of a particle phase and a continuum phase. In the particle phase, each biomass particle falls from the top to the bottom of the thin slice. During the fall, each particle loses mass and volume due to thermal conditions and chemical reactions, e.g., heat transfer, drying, pyrolysis, and/or heterogeneous reactions. The loss mass is released to the bulk phase as an addition to the bulk gas entering the thin slice. Each particle, now with reduced mass and volume, enters the thin slice below with an updated falling velocity, size, and set of compositions (proportions of char, wood, and ash).

The heat transfer to the particle phase from the bulk phase consists of convective and radiative heat transfer. The change of particle temperatures due to heat transfer is expressed in eq E1
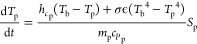
E1where *T*_p_, *T*_b_, *t*, *h*_*c*p_, σ, ϵ, *m*_p_, *c*_*P*p_, and *S*_p_ represent the particle temperature, bulk temperature, time, particle convective heat transfer coefficient, Stephen–Boltzmann constant, emissivity, particle mass, particle heat capacity, and particle surface area, respectively.

**Table 3:** Reaction Kinetic Equations and Parameters for Pyrolysis, Char Heterogeneous Reactions, and Drying^21−24^

descriptor	equation	*A*	*E*_A_ (J mol^–1^)
wood pyrolysis	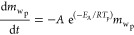 E2	4.4 × 10^9^	1.5 × 10^5^
CO_2_ gasification	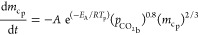 E3	9.1 × 10^6^	1.7 × 10^5^
H_2_O gasification	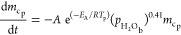 E4	1.7 × 10^3^	1.8 × 10^5^
oxidation	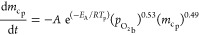 E5	5.3 × 10^5^	1.3 × 10^5^
drying	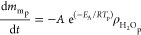 E6	5.1 × 10^9^	88 × 10^3^

**Table 4:** Temperature Kinetic Equations and Parameters for Pyrolysis, Char Heterogeneous Reactions, and Drying

descriptor	equation	Δ*H*_rx,298K_ (J kg^–1^)
wood pyrolysis	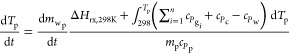 E7	4.2 × 10^5^
CO_2_ gasification	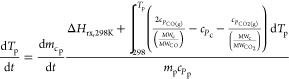 E8	1.4 × 10^7^
H_2_O gasification	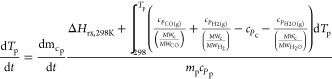 E9	1.1 × 10^6^
oxidation	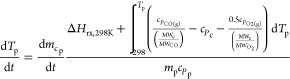 E10	–9.2 × 10^6^
drying	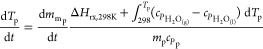 E11	2.4 × 10^6^

Wood, as the main constituent of the particle, is initially pyrolyzed into volatile matter and char according to reaction R1.^20^
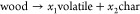
R1

Char heterogeneous reactions are expressed in reactions R2–R4 for CO_2_ gasification, H_2_O gasification, and oxidation, respectively.
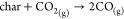
R2
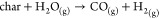
R3
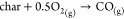
R4

Moisture in the particle evaporates according to reaction R5.
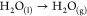
R5The reaction and temperature kinetic equations and parameters for pyrolysis, char heterogeneous reactions, and drying (reactions R1−R5) are listed in Tables 3 and 4.Here, *m*_wp_, *m*_cp_, *m*_mp_, *A*, *E*_A_, *R*, *p*_CO_2_b_, *p*_H_2_Ob_, *p*_O_2_b_, and ρ_H_2_Op_ are the particle wood mass, particle char mass, particle moisture mass, pre-exponential factor, activation energy, gas constant, bulk CO_2(g)_ pressure, bulk H_2_O_(g)_ pressure, bulk O_2(g)_ pressure, and particle moisture density, respectively.Here, Δ*H*_rx,298K_, *c_P_*, and MW are the chemical reaction heat at 298 K, specific heat capacity, and molecular weight, respectively.

The falling velocity of each particle is calculated via eqs E12–E14
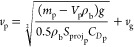
E12
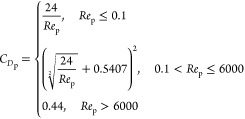
E13
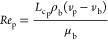
E14where *v*_p_, *C*_*D*p_, *Re*_p_, *V*_p_, ρ_b_, *S*_projp_, *v*_g_, *L*_cp_, *v*_b_, and μ_b_ are the particle velocity, particle drag coefficient, particle Reynolds number, particle volume, bulk density, particle projected area, gas velocity, particle characteristic length, bulk velocity, and bulk viscosity, respectively.

**Figure 4:**
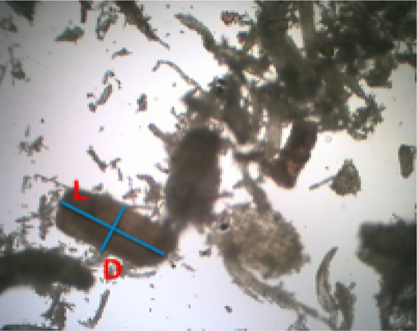
Microscopic images of white wood. L: length of a single particle; D: diameter of single particle.

**Figure 5:**
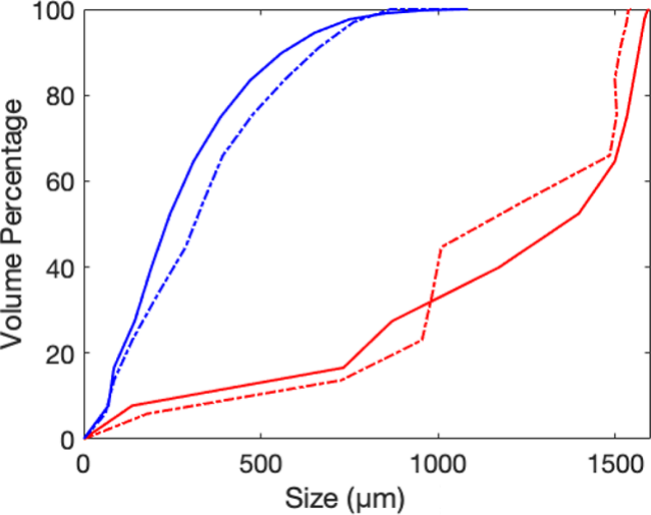
Cumulative particle length and diameter distribution: blue solid line, cylindrical radius of white wood; blue dashed line, cylindrical radius of recycled wood; red solid line, cylindrical length of white wood; red dashed line, cylindrical length of recycled wood.

The values of *V*_p_, *L*_cp_, and *S*_projp_ were determined according to particle shapes. Visual observation, generating several microscopic images of the particles, was conducted to investigate the actual particle shape. The observation revealed that most of the white wood and recycled wood particles were approximately cylindrical (Figure 4). Wood contains a significant quantity of cellulose fibers and lignin as a skeleton holding the structure together. The structure forms parallel alignment of the fibers, resembling long cylinders.^25^ The lengths and diameters of the particles graphically presented in the images were measured using the computer program ImageJ to obtain the particle aspect ratios as a function of particle lengths *L*_p_ (eq E15). The particle lengths were calculated as a function of the particle sizes measured experimentally via eq E16. The experimentally measured particle sizes (*D*_pM_) were as if the particles were spherical. The calculated particle length and diameter distributions are shown in Figure 5.
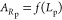
E15
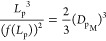
E16

The molar mass of each volatile gas, e.g., CH_4_, CO, CO_2_, H_2_, and H_2_O, produced from pyrolysis was solved via a system of linear equations based on the ultimate analysis data. The mass fraction of each volatile gas was calculated via eq E17
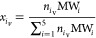
E17where *x*_*i*v_ and *n*_*i*v_ are the mass fraction and molar mass of volatile gas *i*, respectively.

The equations to calculate the molar mass rate profiles of gases both released from the particle phase to the bulk phase and taken by the particle from the bulk phase to the particle phase (*Ḟ*_*i*p_) are listed in Table 5.

**Table 5:** Molar Rates of Gas Released from and Taken by the Particle^a^

		*a*						
reaction	equation	CH_4(g)_	CO_(g)_	CO_2(g)_	H_2(g)_	H_2_O_(g)_	O_2(g)_	N_2(g)_
(R1)	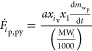 E18	1	1	1	1	1	0	0
(R2)	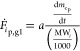 E19	0	2	–1	0	0	0	0
(R3)	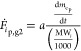 E20	0	1	0	1	–1	0	0
(R4)	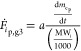 E21	0	1	0	0	0	–0.5	0
(R5)	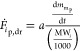 E22	0	0	0	0	1	0	0

a py: pyrolysis; g1: CO_2_ gasification; g2: H_2_O gasification; g3: O_2_ gasification; dr: drying.

The total molar rates of gas *i* released from and taken by an entire particle group of size *j*, *F*_*i*p(*j*)_, are calculated in eq E23.
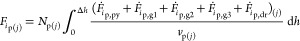
E23

The number of particles in a particle size *j* group, *N*_p(*j*)_, is calculated from the particle length and diameter distribution (Figure 5).

In the continuum phase, the concentrations, temperatures, and velocities of the bulk gas are calculated multiple times due to a series of thermodynamic condition changes.

For step 1, the addition of the released gas to the bulk phase changes the concentrations, *C*_*i*b_, and the temperature of the bulk gas as expressed in eqs E24 and E25. The bulk gas velocity remains unchanged (eq E26).
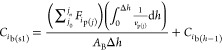
E24
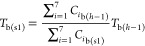
E25
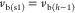
E26where *A*_B_ and Δ*h* represent the burner column cross-sectional area and burner column thin-slice height, respectively.

For step 2, the concentration, temperature, and velocity of the bulk gas are changed due to homogeneous gas reactions (reactions R6–R8), taking into account any gas released from the particle in step 1.
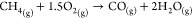
R6
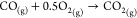
R7
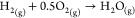
R8

The reaction and temperature kinetic equations and parameters for reactions R6–R8 are expressed in eqs E27–E30 and Table 6.^26,27^
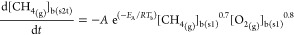
E27
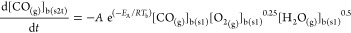
E28
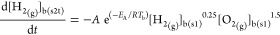
E29
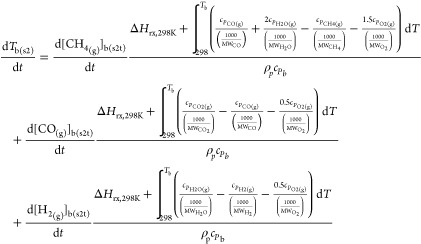
E30

**Table 6:** Reaction and Temperature Kinetic Equations and Parameters for Homogeneous Bulk Gas Reactions

reaction	*A*	*E*_A_ (J mol^–1^)	Δ*H*_rx,298K_ (J mol^–1^)
(R6)	1.6 × 10^10^	2 × 10^5^	–5.2 × 10^5^
(R7)	1.3 × 10^10^	1.8 × 10^5^	–2.8 × 10^5^
(R8)	(3.8 × 10^13^)*T*^–1^	1.7 × 10^5^	–2.4 × 10^5^

The temperature of bulk gas in step 2, *T*_b(s2)_, was obtained from the calculations of eqs E23–E26. The concentration of the bulk gas in step 2, *C*_*i*b(s2)_, was then calculated via an adjustment of temporary bulk gas concentration, *C*_*i*b(s2t)_, using the ideal gas eq E31. The velocity of the bulk gas in step 2 was finally calculated via eq E32.
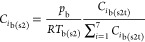
E31
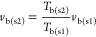
E32

For step 3, the concentrations, temperature, and velocity of bulk gas are modified due to heat loss from the burner to the environment. The heat is consecutively delivered via convection from the bulk gas to the internal surface of the burner, conduction from the internal surface to the external surface of the burner, and convection from the external surface of the burner to the external environment. The convective heat transfer coefficient for the convection from the bulk gas to the internal surface of the burner, *h*_*c*b(s2)_, is calculated via eq E33
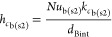
E33where *Nu*_b_, *k*_*c*b_, and *d*_Bint_ represent the bulk Nusselt number, bulk thermal conductivity, and burner internal diameter, respectively.

The wall of the burner is a circular structure made of polycrystalline, high-alumina fibers blended with aluminosilicate fibers and refractory alumina with a 4:1 alumina-to-silica ratio. The thermal conductivity of this alloy, *k*_*w*(s2)_, is calculated via eq E34.
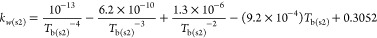
E34

Air is assumed to be the only gas present in the external environment and is assumed to be approximately stagnant. Under these assumptions, the convective heat transfer for the air, *h_e_*, is estimated to be 10.45 W m^–2^ K^–1^. The calculation of the overall burner heat loss coefficient, *U*_*w*(s2)_, is expressed in eq E35
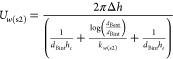
E35where *d*_Bext_ is the burner external diameter. The bulk gas temperature, concentrations, and velocity in step 3 are calculated in eqs E36–E38.
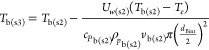
E36
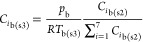
E37
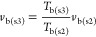
E38

For step 4, in early modeling, the temperature of the gases introduced to the burner was insufficient to initiate thermal conversion (to put it simply, the burner did not ignite). Due to the significant difference between the burner internal diameter and the diameter of the gas introducer at the top of the burner, experimental observations showed that bulk gas with a high temperature was recirculated to the top of the burner and the gas was heated as it was introduced to the burner. This recirculation established steady-state combustion. To simulate this phenomenon and initiate ignition, a small amount of heat (essentially enough to raise the temperature to the ignition point) was “loaned” to the bulk gas at the uppermost section of the burner to initiate ignition in the model. Heat was loaned gradually to the bulk gas until 0.1 m from the top of the burner (eq E39).
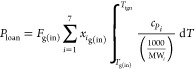
E39

The loaned heat was equally distributed in each individual thin slice (*P*_loanΔ*h*_). The loaned heat, *P*_loan_, was equally gradually removed in each individual subsequent thin slice, *P*_removeΔ*h*_, once the thermal conversion was completed. *F*_g(in)_, *T*_g(in)_, and *T*_ign_ are the inlet gas flow rate, inlet gas temperature, and ignition temperature set as the starting point of biomass thermal conversion, respectively. The temperature of bulk gas in step 4 was obtained by solving *T*_b(s4)_ in either eq E40 or eq E41.
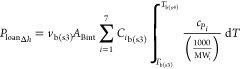
E40
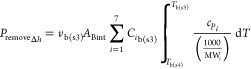
E41

The concentration and velocity of bulk gas in step 4 (eqs E42 and E43) and *T*_b(s4)_ were used as the final properties of bulk gas leaving the thin slice (*C*_*i*b(*h*)_, *v*_b(*h*)_, and *T*_b(*h*)_).
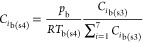
E42
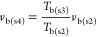
E43

The EFOM separately calculated the fate and occurrence of the AFEs in both the particle phase and bulk phase with the profiles of concentrations (*C*_*i*b_), temperatures (*T*_b_), and velocities (*v*_b_) of the major gases (as discussed above, CH_4_, CO, CO_2_, H_2_, H_2_O, and N_2_) as fixed quantities.

As discussed above, the pyrolysis of a given mass of wood led to the production of a number of AFEs, which were then included in the equilibrium calculations.
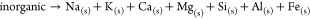
R9

Reaction R9 is a simplification of the decomposition since AFEs are originally not in the form of pure elements.^28^ The initial occurrence of each AFE depends on the AFE cellular locations and interactions with the other AFEs and the organic constituents. The simplification ignores the decomposition heat of the inorganic constituents; however, the decomposition heat value is significantly less than the heat transferred to the particle via oxidation, convection, and radiation. A detailed validation of this assumption has been conducted elsewhere, and it led to a difference in temperature for the particles at <10 K, which was deemed acceptable.^29^

The AFEs formed in reaction R9 were introduced to the chemical equilibrium calculation to obtain the ash-forming compounds (AFCs) formed at *T*_b(*h*)_. All AFCs that, after the equilibrium calculation, were found to be solids or liquids remained within the particle phase, and all gaseous AFCs that were formed left for the continuum phase.

The AFC concentrations in the continuum (i.e., gas plus any aerosols formed) phase similarly change in a series of steps during each time step.

For step 1, the addition of the released gaseous AFCs (GAFC) to the continuum phase changes the concentrations of bulk AFCs (eq E44).
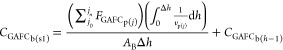
E44

The concentrations of bulk solid and liquid aerosols (SAFC and LAFC) entering the thin slice from above remained unchanged (eqs E45 and E46).
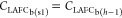
E45
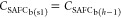
E46

For step 2, the concentrations of bulk ash-forming elements in step 1 were used as the input to the chemical equilibrium calculation at *T*_b(*h*)_ to obtain the concentrations of liquid and solid aerosols (i.e., AFCs), e.g., *C*_GAFCb(s2)_, *C*_LAFCb(s2)_, and *C*_SAFCb(s2)_. Here, the concentrations of aerosols can change; this is the primary step where they are formed.

For step 3, the concentrations of gaseous AFCs were adjusted using eq E47 to ensure that the pressure remained at 1 bar; the total quantities of solid and liquid aerosols do not change from step 2 to step 3 (eqs E48 and E49).
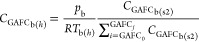
E47
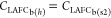
E48
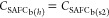
E49where *p*_b_ is the bulk pressure.

## Results and Discussion

### Entrained-Flow Combustion Model

**Figure 6:**
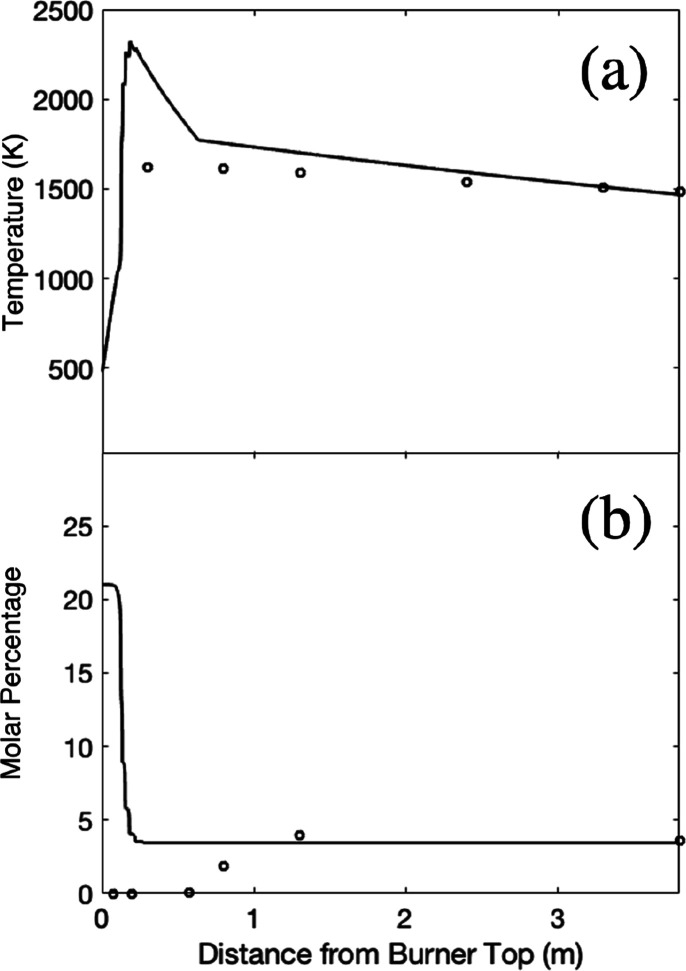
Profiles of (a) bulk temperature and (b) bulk O_2(g)_ molar fraction: solid lines, ECM; open circles, experiment at the PACT facility.

The temperature and dry O_2(g)_ molar percentage profiles measured in the burner combusting white wood with air were compared with the profiles generated with ECM (Figure 6). The comparison shows that the ECM profile results were well validated with the measured profiles. However, the peak temperature (±0.25 m from burner top) shown in the ECM bulk temperature profile was unable to be validated. The combustion occurred within 0.5 m from the burner top. The feed introducer diameter is far shorter than the burner internal diameter. When both fuel and air entered the burner, their combined velocity was reduced due to cross-sectional area expansion. The slower flow promoted both fuel and air to move away from the burner centerline. Due to less fuel at the burner centerline, the combustion was thought to occur primarily near burner walls within the burner’s uppermost section, causing lower centerline temperatures than near-wall temperatures. The occurrence of the oxidation reactions near the burner wall distributed all the O_2_ to that location and left the burner centerline free of O_2(g)_. Since ECM works as a one-dimensional model, not accounting radial thermal distribution, and the temperature probes were installed to measure several positions within the burner centerline, the discrepancy between the modeled temperature profiles and the measured temperature profiles are expected within 0.5 m from the burner top.

### EFOM: Alkali Metals (Figures 7 and 8)

**Figure 7:**
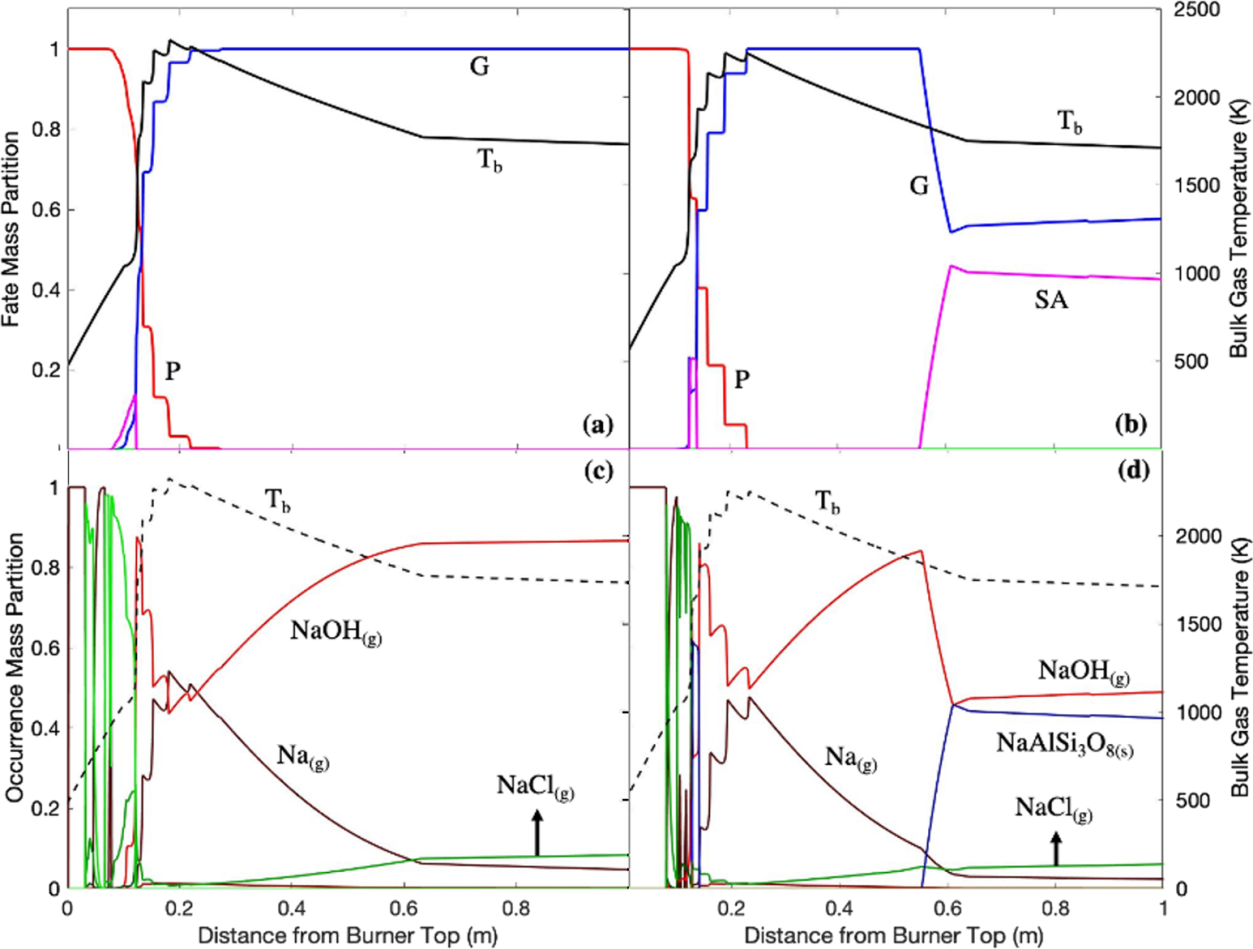
Sodium phase profile in (a) white wood combustion and (b) recycled wood combustion with air; sodium occurrence profile in (c) white wood combustion and (d) recycled wood combustion with air. P: particle; G: gas; LA: liquid aerosol; SA: solid aerosol; *T*_b_: bulk temperature.

**Figure 8:**
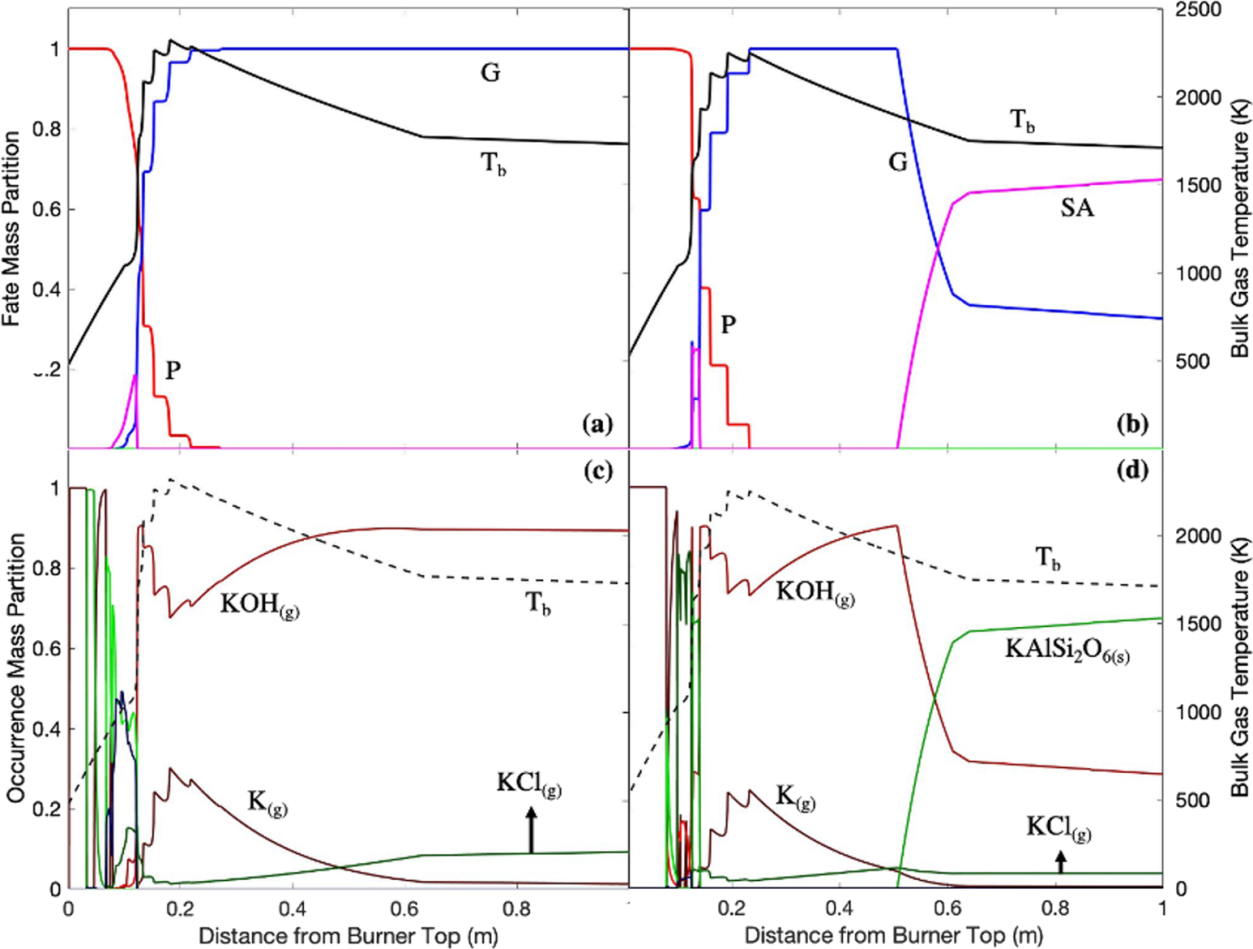
Potassium phase profile in (a) white wood combustion and (b) recycled wood combustion with air; potassium occurrence profile in (c) white wood combustion and (d) recycled wood combustion with air. P: particle; G: gas; LA: liquid aerosol; SA: solid aerosol; *T*_b_: bulk temperature.

Figures 7 and 8 present the fate and occurrence of both sodium and potassium along the burner as the bulk temperatures change. Each figure exhibits different behaviors between the profiles before the peak temperature (0.2 m) and after the peak temperature. The occurrence profiles were simpler after the peak temperature since only a relatively small number of compounds exist within a broad height range. In addition, the evaluation and comparison of the theoretical predictions with experimental measurements were easier since the calculated and measured temperatures for the continuum phase were similar after the peak in temperature. The occurrence profiles before peak temperature, however, were difficult to evaluate due to significant changes in composition within a small number of slices. Nevertheless, the actual quantities of trace elements released are small because the temperatures before the peak temperature are low so that the biomass does not release any metals to the bulk phase until the temperature is close to the peak temperature. This also applies to the other elements.

Figure 7 shows that once the peak temperatures were achieved (0.2 m), all the alkali metals were devolatilized from the particles since both sodium and potassium were very volatile at relatively low temperatures. The alkali metals were released mostly as alkali metal hydroxides and chlorides (KOH_(g)_, NaOH_(g)_, KCl_(g)_, and NaCl_(g)_) and as small quantities of K_(g)_ and Na_(g)_ shortly before the bulk temperature profiles reached the peak temperature, although the majority of the metals were present as hydroxides owing to reactions with moisture (reactions R10–R13).^30−32^ It is reasonable to use an equilibrium model here since reactions R12 and R13 are frequently experimentally found to be in equilibrium due to instantaneous forward and backward reaction rates at high temperatures.^33^
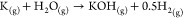
R10
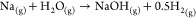
R11
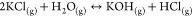
R12
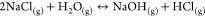
R13

Above and toward 2000 K, alkali metal hydroxides have a tendency to decompose back into pure elements due to extreme heat as the occurrence profiles of Na_(g)_ and K_(g)_ spike at the peak temperature.^34^

Below 0.5 m, alkali aluminum silicates (NaAlSi_3_O_8(s)_ and KAlSi_2_O_6(s)_) were formed in recycled wood combustion. With the absence of alkali aluminum silicates in white wood combustion, the difference in occurrence indicates that the larger amount of alkali metals released from recycled wood was sufficient to undergo a series of complex reactions with silicon and aluminum oxides to form NaAlSi_3_O_8(s)_ and KAlSi_2_O_6(s)_.^35^ NaAlSi_3_O_8(s)_ has frequently been found in the fly ash of combustion of solid fuel with high sodium content.^36^ In a commercial boiler, KAlSi_2_O_6(s)_ has frequently been found in the refractory materials growing on the surface of superheaters along with other alkali aluminum silicates, e.g., NaAlSi_3_O_8(s)_ and KAlSiO_4(s)_.^37^ The reaction of alkali metals with silicon and aluminum oxides might have occurred via aerosol collisions prior to deposition on superheater surfaces and, subsequently, within the refractory materials (reaction R14).^38^
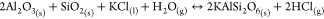
R14

Alkali chlorides (NaCl_(g)_ and KCl_(g)_) were both formed with small proportions in both white wood and recycled wood combustion. The devolatilized alkali metals may have reacted with chlorine once released from the particle phase to alkali chlorides.^39^ The alkali chlorides might have also reacted with the volatilized sulfur (SO_2(g)_) to form a minor amount of alkali sulfates (reactions R15 and R16). However, both reactions R15 and R16 are significantly slow and require adequate reaction times for high conversion, e.g., during the growth of the fouling scale initiated by alkali chloride layers.^40^
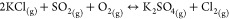
R15
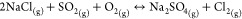
R16

**Figure 9:**
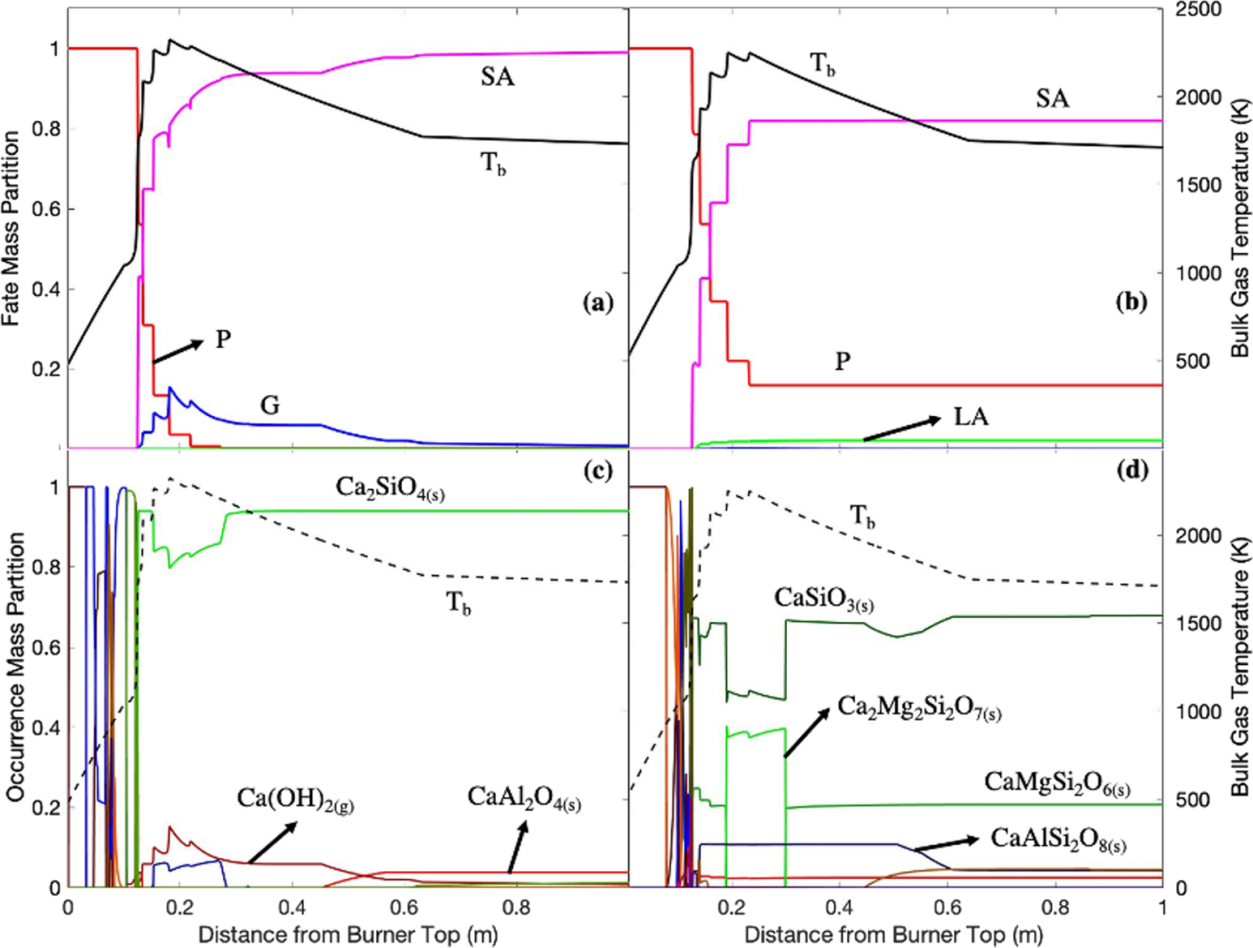
Calcium fate profiles in (a) white wood combustion and (b) recycled wood combustion with air; calcium bulk occurrence profiles in (c) white wood combustion and (d) recycled wood combustion with air. P: particle; G: gas; LA: liquid aerosol; SA: solid aerosol; *T*_b_: bulk temperature.

**Figure 10:**
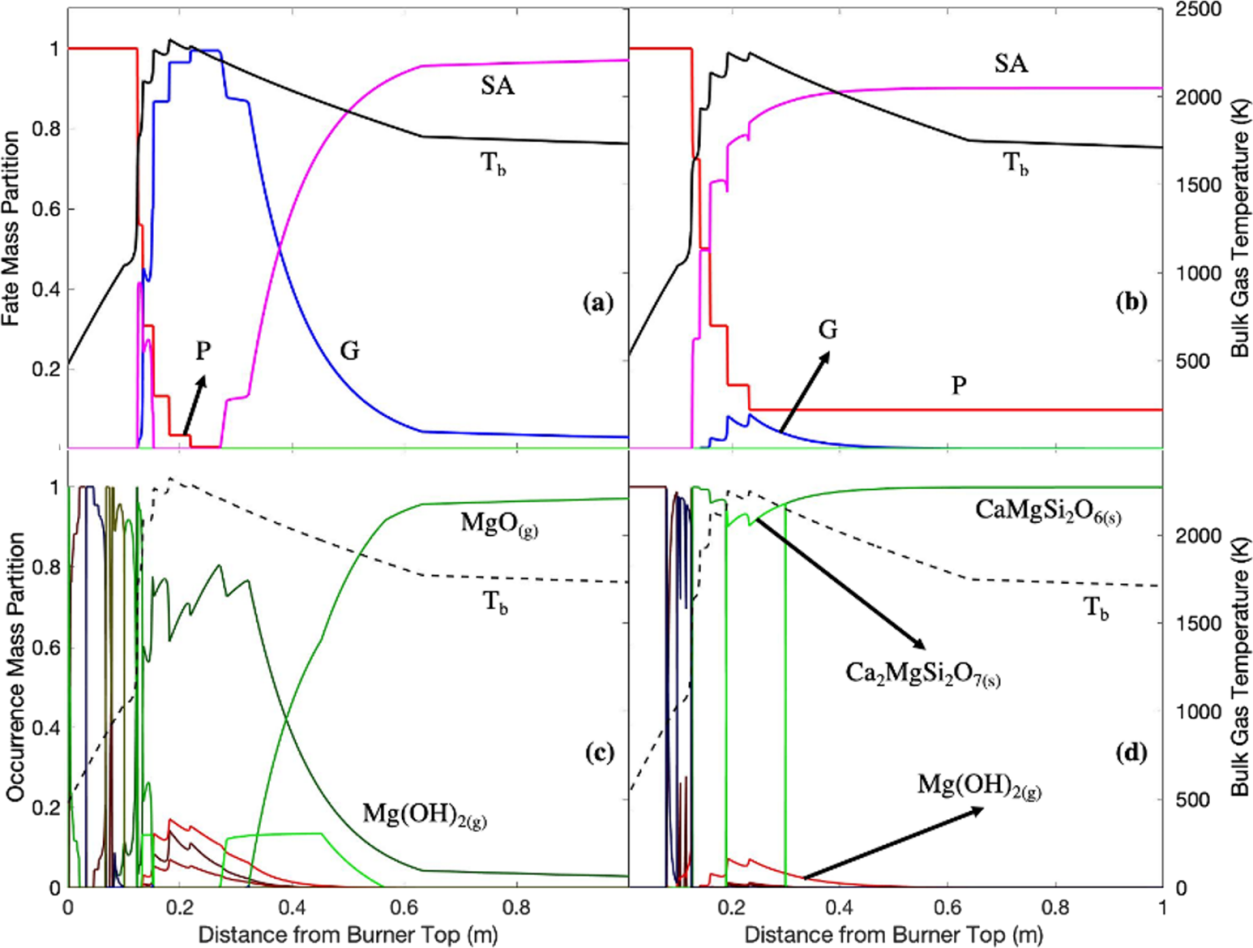
Magnesium fate profiles in (a) white wood combustion and (b) recycled wood combustion with air; magnesium species present in (c) white wood combustion and (d) recycled wood combustion with air. P: particle; G: gas; LA: liquid aerosol; SA: solid aerosol; *T*_b_: bulk temperature.

### EFOM: Alkaline Earth Metals (Figures 9 and 10)

The alkaline earth metals were unexpectedly very volatile and released almost completely (±0.1 m) from the particle phase in white wood combustion, probably due to less alkaline earth metal quantities in white than in recycled wood.^41^ When released, the alkaline earth metals, primarily calcium, exhibited high tendencies to react with silicon to form solid alkaline earth metal oxide aerosols.^42^

Ca_2_SiO_4(s)_ was formed with the highest proportion as the result of calcium oxide reaction with silicon oxide in white wood combustion (reaction R17). The reaction to form Ca_2_SiO_4(s)_ is favored at temperatures from 1143 to 1873 K.^43,44^
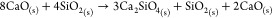
R17

Despite a substantial amount of carbon in the form of CO_2_ in the continuum phase, the formation of CaCO_3(s)_ did not occur due to the very high temperatures, and a small amount of Ca(OH)_2(g)_ was formed in white wood combustion.^45,46^

Calcium formed a significant number of silicate aerosols with magnesium, aluminum, and silicon in the combustion of recycled wood. For white wood, the quantity of calcium was much higher than the quantity of silicon, while recycled wood featured similar quantities of both elements. The formation of CaSiO_3(s)_ is favored when the CaO-to-SiO_2_ ratio is at around 1.^47^ CaSiO_3(s)_ is also formed via Ca_2_SiO_4(s)_’s further reaction with SiO_2(s)_ (reaction R18).^48^
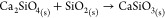
R18

CaMgSi_2_O_6(s)_ is formed in a lower proportion but still has unclear mechanisms of formation. Despite this, CaMgSi_2_O_6(s)_ was evidently found in fir wood combustion fly ash.^49^

The occurrence of magnesium in white wood combustion was primarily Mg(OH)_2(g)_ and MgO_(s)_. Magnesium is mainly devolatilized (± 0.1 m) as Mg(OH)_2(g)_. The formation of Mg(OH)_2(g)_ at around the peak temperature (0.1–0.3 m) was likely due to magnesium’s reaction with moisture at a very high temperature.^50^ As the temperature gradually decreases (>0.3 m), Mg(OH)_2(g)_ decomposed into MgO_(g)_ (reaction R19).^51^
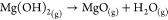
R19

In recycled wood combustion, the occurrence of magnesium was dominated by Ca–Mg silicates. Once released from the particle phase, magnesium formed CaMgSi_2_O_6(s)_. CaMgSi_2_O_6(s)_ was converted to Ca_2_MgSi_2_O_7(s)_ at around the peak temperature (0.19 m) and regained its initial form when the bulk temperature decreased (0.3 m) due to the stability of CaMgSi_2_O_6(s)_ at low temperatures.^52^

The alkaline earth metals were left in the particle phase at a significant proportion at around 20 and 10% of the total initial quantity of calcium and magnesium, respectively, in recycled wood combustion. High quantities of alkaline earth metals in the solid fuel might have increased the retention of the alkaline earth metals in the particle phase due to formation of complex silicates because of reactions with O_2_ penetrating during char oxidation. CaSiO_3(s)_ might have been formed via calcium reactions with silicon and O_2_.^53^ The selectivity toward CaSiO_3(s)_ formation may have also been suppressed due to the interference of magnesium and aluminum, forming the other silicates, e.g., Ca_3_MgSi_2_O_8(s)_ and Ca_2_Al_2_SiO_7(s)_.^54,55^

**Figure 11:**
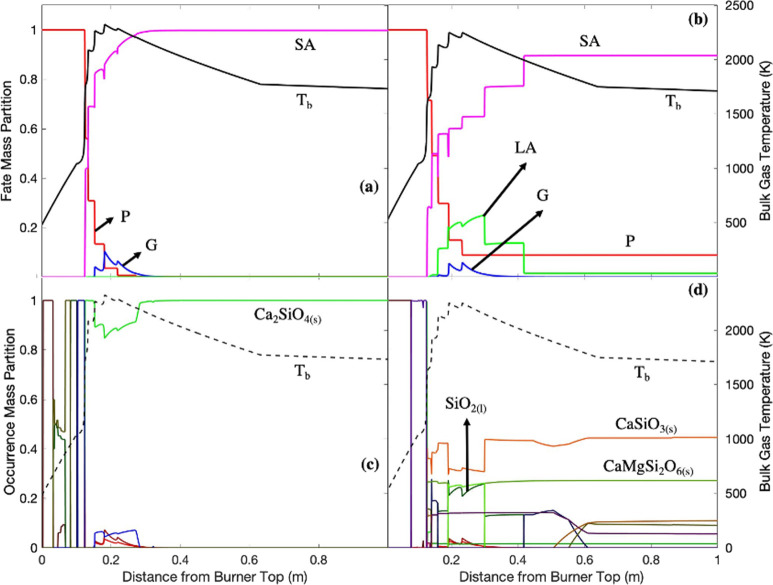
Silicon fate profiles in (a) white wood combustion and (b) recycled wood combustion with air; silicon bulk occurrence profiles in (c) white wood combustion and (d) recycled wood combustion with air. P: particle; G: gas; LA: liquid aerosol; SA: solid aerosol; *T*_b_: bulk temperature.

### EFOM: Silicon (Figure 11)

The release of silicon (±0.1 m) exhibited similar behavior to the release of calcium in both white wood and recycled wood combustion. This similarity was probably due to silicon affinity with calcium in forming calcium silicates as previously shown in Figure 9. Both silicon and calcium were released from the particle phase in the forms of Ca_2_SiO_4(s)_ and CaSiO_3(s)_ in the combustion of white wood and recycled wood, respectively.

When compared to white wood combustion, the very distinctive behavior of silicon in recycled wood combustion was the formation of a substantial fraction of liquid compounds at around the peak temperature (0.19–0.3 m). The combustion of recycled wood might have promoted the formation of SiO_2(l)_ since recycled wood contains a greater quantity of silicon than white wood. The formation of SiO_2(l)_ has been previously described as SiO_2(g)_ supersaturation.^56^ Silicon was likely volatilized as SiO_(g)_, subsequently reacting with O_2(g)_ (reaction R20). SiO_2_(g) might have also been formed via SiO_(g)_ heterogeneous nucleation with CO_2(g)_ (reaction R21).
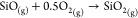
R20
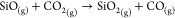
R21

In the case of combustion of recycled wood, the saturation of SiO_2(g)_ might have been established via the breakdown of CaSiO_3(s)_ in the very hot environment at around the peak temperature into other calcium silicate compounds and SiO_2(g)_. The saturation of SiO_2(g)_ may have led to the formation of calcium silicates when exposed to calcium compounds and, further, to the nucleation SiO_2(g)_ into SiO_2(l)_ (reaction R22).
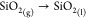
R22

SiO_2(l)_ later diminishes, and CaSiO_3(s)_ again became the dominant species below 2000 K. A tiny quantity of CaSiTiO_5(l)_ was also found and has previously been found to be present in such systems.^57^

The remaining silicon left in the particle phase in recycled wood exhibited a similar occurrence profile to the remaining calcium (Figure 9). Silicon’s strong affinity with calcium promoted the formation of calcium silicate compounds, e.g., CaSiO_3(s)_, Ca_3_MgSi_2_O_8(s)_, and Ca_2_Al_2_SiO_7(s)_.

**Figure 12:**
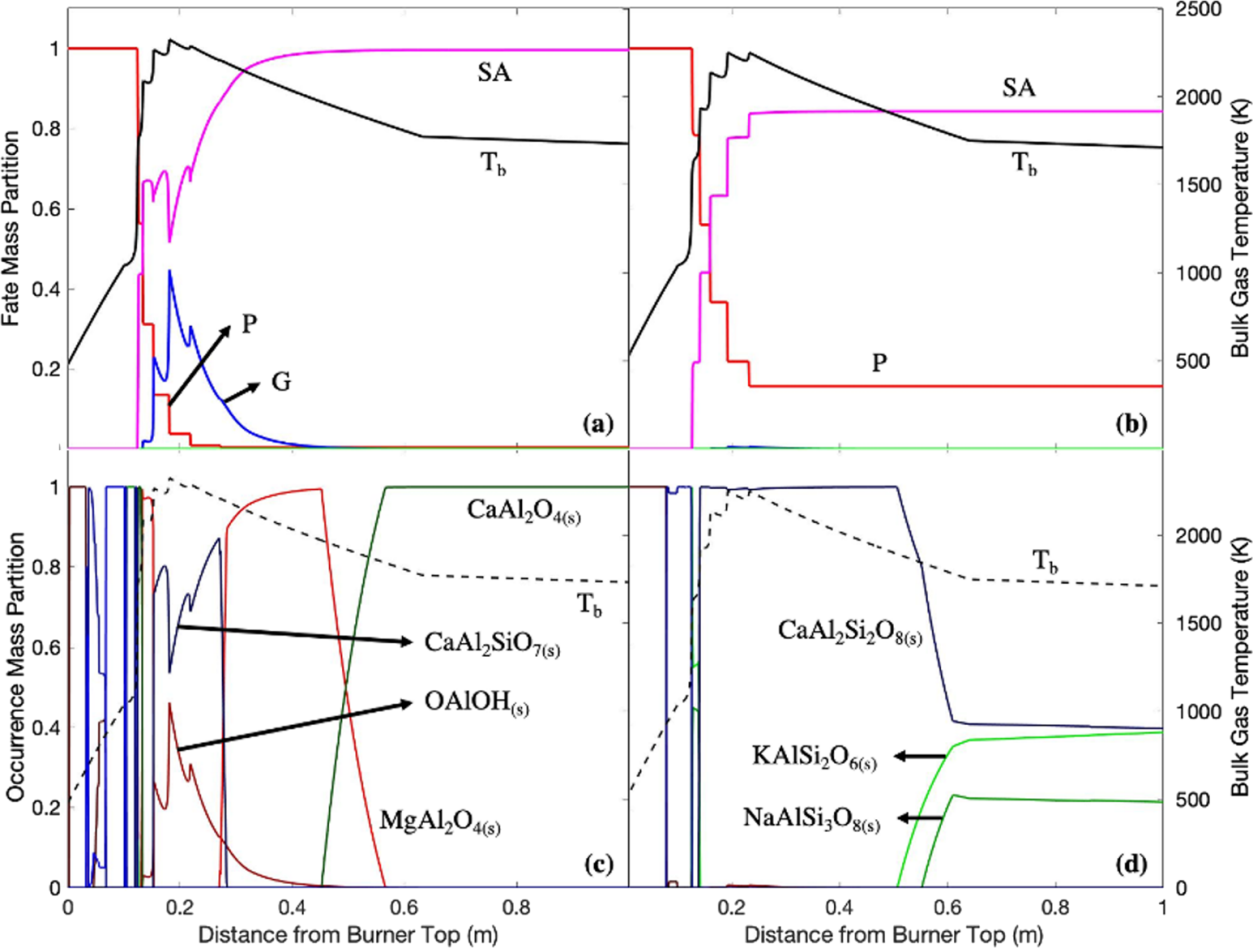
Aluminum fate profiles in (a) white wood combustion and (b) recycled wood combustion with air; aluminum bulk occurrence profiles in (c) white wood combustion and (d) recycled wood combustion with air. P: particle; G: gas; LA: liquid aerosol; SA: solid aerosol; *T*_b_: bulk temperature.

### EFOM: Aluminum (Figure 12)

Aluminum was entirely released (±0.1 m) from the particle phase in white wood combustion, while almost 20% of the total initial aluminum remains in the particle phase in recycled wood combustion. The remaining aluminum in recycled wood combustion indicated aluminum affinity with the remaining silicon and calcium in forming aluminosilicate compounds.

In white wood combustion, aluminum was released in significant quantities as AlO_(g)_. At around the peak temperature, aluminum was in the form of OAlOH_(g)_ and Ca_2_Al_2_SiO_7(s)_. OAlOH_(g)_ was formed via the AlO_(g)_ reaction with moisture (reaction R23).
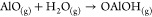
R23

At bulk temperatures close to the peak temperature, OAlOH_(g)_ was found to be more prevalent, and at lower temperatures, the strong interactions of calcium and silicon with aluminum preferentially formed compounds such as Ca_2_Al_2_SiO_7(s)_. Since recycled wood contains more AFEs, the formation of CaAl_2_Si_2_O_8(s)_ was preferred in this case. CaAl_2_Si_2_O_8(s)_ might have been formed via further reaction of Ca_2_Al_2_SiO_7(s)_ with oxidized silicon and aluminum (reaction R24).^58^
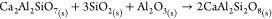
R24

As bulk temperatures dropped (0.28–0.45 m) in white wood combustion, MgAl_2_O_4(s)_ was formed along with MgO_(s)_ (Figure 10c). The coexistence of MgAl_2_O_4(s)_ and MgO_(s)_ may have been due to their stability in forming a solid solution at temperatures above 1773 K.^59^ When approaching the end of step 3 (0.45–0.56 m), the bulk temperatures were low enough to allow MgAl_2_O_4(s)_ decomposition and CaAl_2_O_4(s)_ formation via nucleation.^60^

At around 0.5 m from the top of the burner in recycled wood combustion, low bulk temperatures reduced the proportions of CaAl_2_Si_2_O_8(s)_ and alkali aluminosilicates, e.g., KAlSi_2_O_6(s)_ and NaAlSi_3_O_8(s)_. The selectivity toward alkali aluminosilicate formation was probably due to higher silicon and alkali metal quantities in recycled wood than in white wood.

In the bottom ash in recycled wood combustion, aluminum remains as Ca_2_Al_2_SiO_7 (s)_. Although the mechanism of Ca_2_Al_2_SiO_7(s)_ formation in the bottom ash is still unclear, the formation might have occurred via calcium, silicon, and aluminum chemical interactions enhanced with the larger quantities of such elements in recycled wood than in white wood.

**Figure 13:**
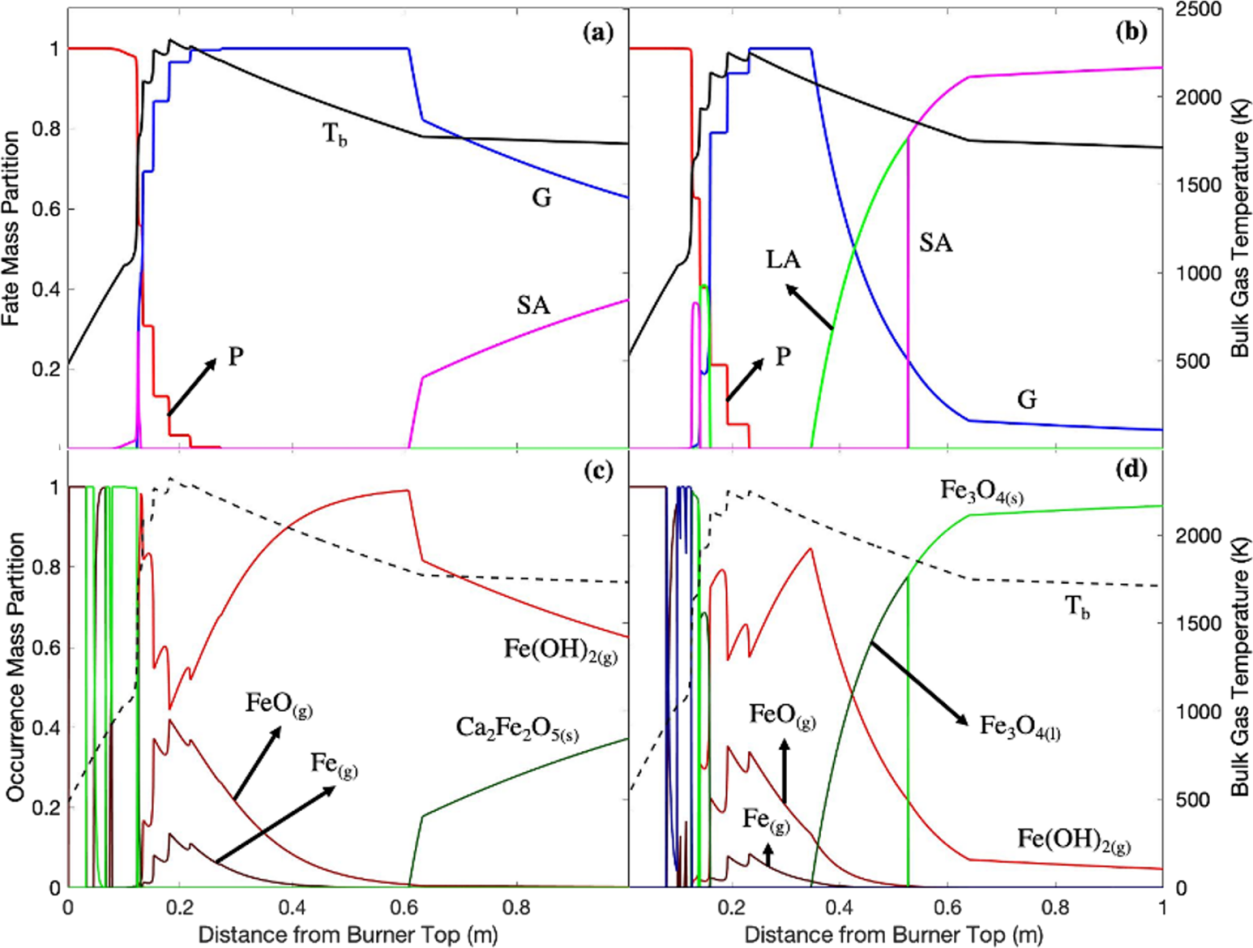
Iron fate profiles in (a) white wood combustion and (b) recycled wood combustion with air; iron bulk occurrence profiles in (c) white wood combustion and (d) recycled wood combustion with air. P: particle; G: gas; LA: liquid aerosol; SA: solid aerosol; *T*_b_: bulk temperature.

### EFOM: Iron (Figure 13)

The entirety of the iron was released (±0.1 m) from the particle phases in both white wood and recycled wood combustion. Iron might have been devolatilized in to the particle phase as Fe_(g)_. However, the primarily form of iron during the release was Fe(OH)_2(g)_ due to Fe_(g)_ reaction with moisture (reaction R25).^61^ The remaining unreacted Fe_(g)_ was left along with FeO_(g)_ in small quantities. It was also possible that FeO_(g)_ could react with moisture to form Fe(OH)_2_.
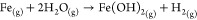
R25

At peak temperatures (± 0.2 m), the proportion of Fe(OH)_2(g)_ decreased as the occurrence partition of Fe_(g)_ and FeO_(g)_ increased. Very high temperatures might have promoted Fe(OH)_2(g)_ decomposition via the reverse reaction of R25. As bulk temperatures gradually decreased (>0.2 m), the proportion of Fe(OH)_2(g)_ increased and the quantities of Fe_(g)_, and FeO_(g)_ decreased to zero.

However, while the Fe(OH)_2(g)_ proportion increased and remained stable (>0.2 m) in white wood combustion, Fe(OH)_2(g)_ was converted to Fe_3_O_4(l)_ (0.35–0.53 m) in recycled wood combustion (reaction R26).^62^
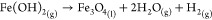
R26

The conversion of Fe(OH)_2(g)_ to Fe_3_O_4(l)_ is known as the Schikorr reaction and might have been due to a greater quantity of iron in the recycled wood than in the white wood. Fe_3_O_4(l)_ was turned into Fe_3_O_4(s)_ (>0.52 m) when bulk temperatures dropped below 1870 K.^63^

### ICP Measurement and Validation

**Figure 14:**
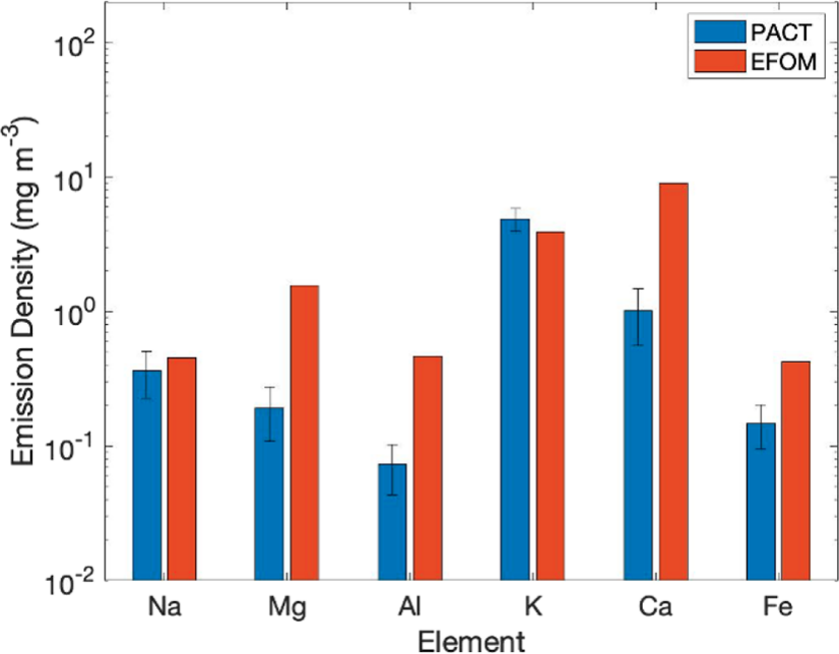
Densities of AFEs leaving the burner in white wood combustion. Error bar: measurement sensitivity range.

The concentrations of AFEs leaving the burner were compared with the AFE concentrations measured using ICP. This would be expected to pick up species in both the gas and aerosol phases. Figure 14 shows the comparison for white wood combustion and shows that the AFE concentrations in uncooled combustion gases are somewhat validated by the ICP measurement results. However, certain limitations promote disagreement between the measurement and the model results. The comparison shows reasonable agreement for alkali metals but higher estimated concentrations for alkaline-earth metals, silicon, aluminum, and iron from the model.

Compared with the alkaline-earth metals, the alkali metals are extremely volatile. As seen in Figures 7a and 8a, no alkali metals remain in the particles. Since the fate and occurrence calculations are based on the minimization of the Gibbs free energy change on which not only temperatures and pressures but also chemical compositions have an effect, the significantly lower alkali metal molar quantities might not permit the formation of alkali aluminosilicates as seen in Figures 7c and 8c. Instead, the released silicone and aluminum are prone to form calcium aluminosilicates with calcium, e.g., Ca_2_SiO_4(s)_, as seen in Figures 9c and 12c. Since Fe_3_O_4(s)_ does not exist in white wood combustion (Figure 13), Fe_3_O_4(s)_ might be formed via the Schikorr reaction at lower bulk temperatures at the lower reaches of the burner.

Unlike the released alkali metal species, which are gaseous, the calcium aluminosilicates are solid aerosols, which have the tendency to form larger-size solid aerosols at relatively low temperatures. The formation of larger-size solid aerosols was promoted via aerosol coagulation. Some compounds might form large growing aerosol particles via nucleation and condensation or recondense on the existing ash particles. SiO_2(s)_, for instance, condenses on the surface of growing Ca_2_SiO_4(s)_ aerosols. The condensation could promote a solid-state reaction converting Ca_2_SiO_4(s)_ into CaSiO_3(s)_ (reaction R27). The nucleated aerosol particle sizes could further increase via aerosol particles coagulation with other aerosol particles or residual particles.
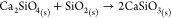
R27

Large aerosol sizes reduce aerosol selectivity to escape the burner and enhance aerosol probability to be retained at burner bottom. The formation of large particles containing Si, Al, Fe, and Mg via coagulation, aerosol growth, or condensation on ash (neither of which are accounted in the model and would be observed by ICP) is likely the reason for the discrepancy between the modeled results and the results from the ICP.

AFEs are distributed originally into several forms in wood, namely, water-soluble salts, organically associated metal ions, included minerals, and excluded minerals. Water-soluble salts are mostly AFE sulfates, chlorides, and phosphates dissolved in pore moisture. Some AFEs are also ionically bonded with organics. AFE interactions among themselves also allow formation of various minerals either distributed within organic matrices or excluded from organic matrices entirely. As the AFE with one of the highest concentrations in wood, calcium and silicon are often trapped as (Ca,Mn)C_2_O_4_·2H_2_O(s) and SiO_2(s)_ within organic matrices. SiO_2(s)_ along with other minerals, e.g., CaAl_2_Si_2_O_8(s)_, Al_2_Si_2_O_5_(OH)_4(s)_, etc., is also clumped as minerals excluded from organic matrices.^28^ AFEs in both excluded and included minerals are more likely to be retained in residual particles collected at the bottom of the burner since minerals have worse volatilization behavior than water-soluble salts and organics, although equilibrium-based models such as those developed here cannot account for such kinetic limitations.

## Concluding Remarks

The integrated ECM and EFOM were designed to predict and evaluate the fate and partition of ash-forming elements in solid fuel combustion. The predicted fate and partition of each ash-forming element were qualitatively compared with experimental results. The comparison exhibited that the experimental findings have some areas of agreement with the modeled results. However, some elements (Fe, Mg, Ca, and Al) were not validated well by experimental measurements. The predicted concentrations of several AFEs overestimated the ICP readings due to the choice of an equilibrium-based model and the likelihood that aerosols forming would in reality recondense on ash particles and hence be removed from the bulk phase, which was measured by the ICP.

The integration of the combustion and equilibrium models developed here appears to be reliable yet still requires improvement, particularly to consider the kinetics of ash-forming element release, which are treated crudely here. Such improvement will require the mathematical development of nucleation and coagulation calculations and comprehensive measurements of the initial forms of the ash-forming elements together with kinetic expressions for their production.

## Notes

Ash-forming elements: chemical elements of sodium, potassium, calcium, magnesium, silicon, aluminum, and iron. Bulk phase: see continuum phase. Continuum phase: burner internal environment, not including solid fuel. Fate: states of elements, e.g., remained in residual particles, flowing in the continuum phase as gases, and flowing in the continuum phase as aerosols (see continuum phase). Major gases: chemical compounds of methane, carbon monoxide, carbon dioxide, hydrogen, steam, oxygen, and nitrogen. Minor species: any chemical compounds containing ash-forming elements (see ash-forming elements). Occurrence: chemical compound forms of elements. Trace species: any chemical compounds containing trace elements, e.g., chemical elements of arsenic, cadmium, copper, cobalt, chromium, nickel, lead, zinc, etc.

## Nomenclature

*A*: pre-exponential factor
*A*_B_: burner internal cross-sectional area (m^2^)
*A_R_*: aspect ratio
*C*: concentration (mol m^–3^)
*C_D_*: drag coefficient
*c_P_*: specific heat capacity (J kg^–1^ K^–1^)
*D*: diameter (m)
*d*_Bext_: burner external diameter (m)
*d*_Bint_: burner internal diameter (m)
*E*_A_: activation energy (J mol^–1^)
*F*: released/taken gas molar rate for an individual particle size cluster (mol s^–1^)
*Ḟ*: released/taken gas molar rate for an individual particle (mol s^–1^)
*g*: Earth’s gravitational acceleration (m s^–2^)
*h*: height (m)
*h_c_*: convective heat transfer coefficient (W m^–2^ K^–1^)
*h_e_*: Earth’s atmosphere convective heat transfer coefficient (W m^–2^ K^–1^)
*k_c_*: thermal conductivity (W m^–1^ K^–1^)
*k_w_*: burner wall thermal conductivity (W m^–1^ K^–1^)
*L*: length (m)
*L*_c_: characteristic length (m)
*m*: mass (kg)
*M*W: molecular weight (g mole^–1^)
*Nu*: Nusselt number
*P*: heat rate (W)
*p*: pressure (Pa)
*R*: gas constant (8.314 J mol^–1^ K^–1^)
*Re*: Reynold number
*S*: surface area (m^2^)
*S*_proj_: projected surface area (m^2^)
*T*: temperature (K)
*t*: time (s)
*T*_e_: Earth’s atmosphere temperature (K)
*U_w_*: burner overall heat transfer coefficient (W m^–2^ K^–1^)
*V*: volume (m^3^)
*v*: velocity (m s^–1^)
*x*: fraction
**Greek Letters**
Δ*H*_rx,298K_: chemical reaction heat at 298 K (J kg^–1^)
ϵ: emissivity
μ: viscosity (Pa s)
ρ: density (kg m^–3^)
σ: Stephen–Boltzmann constant (5.670374419 × 10^–8^ W m^–2^ K^–4^)
**Subscripts**
b: bulk
c: char
g: gas
g1: CO_2(g)_ gasification
g2: H_2_O_(g)_ gasification
g3: O_2(g)_ gasification/combustion
GAFC: gaseous ash-forming species
*h*: burner thin slice index
*i*: species *i*
ign: ignition
in: inlet
*j*: particle size cluster *j*
LAFC: liquid ash-forming species
loan: loaned to the burner
M: measured value
p: particle
py: pyrolysis
remove: removed from the burner
s1: step 1
s2: step 2
s3: step 3
s4: step 4
SAFC: solid ash-forming species
v: volatile
w: wood
Δ*h*: individual burner thin slice
